# Proton pump inhibitors and gastrointestinal symptoms among patients with COVID-19 infection

**DOI:** 10.1080/07853890.2024.2355581

**Published:** 2024-06-01

**Authors:** Hafez Al-Momani, Iman Aolaymat

**Affiliations:** a Department of Microbiology, Pathology and Forensic Medicine, Medical School, The Hashemite University, Zarqa, Jordan; b Department of Anatomy, Physiology and Biochemistry, Medical School, The Hashemite University, Zarqa, Jordan

**Keywords:** Covid-19, gastrointestinal system, COVID-19 manifestation, proton pump inhibitors

## Abstract

**Introduction:**

The administration of proton pump inhibitors (PPIs) is anticipated to elevate an individual’s susceptibility to enteric infections as a result of altering the gut flora. The influence of PPIs on the clinical manifestation of severe acute respiratory syndrome coronavirus 2 (SARS-CoV-2) is still uncertain. This study aims to investigate the impact of PPI usage on the clinical manifestation of COVID-19, namely its gastrointestinal symptoms.

**Methods:**

This is a cross-sectional cohort study involving COVID-19 patients. Patients were interviewed using a predesigned questionnaire that asked about their demographics, clinical manifestations of COVID-19 infection, and the extent and type of PPIs in use. PPI usage was confirmed by reviewing patients’ electronic medical records. The primary outcome was to establish any association between the use of PPI and the symptoms and clinical presentation of COVID-19.

**Results:**

Out of a total of 254 participants, 69 (27.2%) were considered PPI users. Patients who were on PPI medications reported a significantly lower rate of myalgia (27.5% vs 51.9%; *p* = 0.0006) and heartburn (5.7% vs 15.6%; *p* = 0.03) but had a significantly higher rate of abdominal pain (27.5% vs 13.5%; *p* = 0.001) and diarrhoea (28.9% vs 14.5%, *p* = 0.02) when compared to those who were not using PPIs. Patients on PPIs were also shown to have significantly higher odds of developing diarrhoea (OR 2.0, 95% CI: 1.08 to 3.93, *p* = 0.02) and abdominal pain (OR 2.0, 95% CI: 1.22 to 3.93, *p* = 0.03), but a lower risk of developing myalgia (OR 0.5, 95% CI: 0.3 to 0.9, *p* = 0.02) when compared to non-PPI users.

**Conclusion:**

This study shows that the use of PPIs could impact COVID-19 clinical presentation toward more gastrointestinal manifestations. Further studies investigating the link between other acid suppression medications and COVID-19 manifestations and severity should be carried out.

## 1. Introduction

The severe acute respiratory syndrome coronavirus 2 (SARS-CoV-2) has been responsible for the world’s greatest pandemic since the flu of 1918 [1]. The repercussions of the resulting coronavirus disease 2019 (COVID-19) are still being felt across the globe today and it remains the number one public health issue which various governments are still grappling with.

COVID-19 mainly manifests through respiratory symptoms such as cough and shortness of breath [2] but there is increasing evidence that COVID-19 is a multi-system disease. Studies have shown that COVID-19 patients also exhibit non-respiratory symptoms, particularly symptoms affecting the gastrointestinal (GI) system such as diarrhoea, low appetite, and nausea [3,4]. Several studies have found SARS-CoV-2 RNA in the stool of infected patients [5], and with angiotensin-converting enzyme 2 (ACE-2) serving as the viral receptor, it was found to be highly expressed in the GI tract, suggesting that the SARS-CoV-2 also affects the digestive system [6]. Another study also showed that about 20% of COVID-19 patients may exhibit one or more GI symptoms such as diarrhea, vomiting, and abdominal pain [3].

Some of the most common GI diseases that people suffer from are acid-related GI diseases. These include gastroesophageal reflux disease (GERD), esophagitis, and peptic ulcer disease (PUD), which affects about 20% to 30% of adults [7,8]. Proton pump inhibitors (PPIs) are potent acid-lowering medications and are normally prescribed by doctors as a primary defense against acid-related GI diseases [9,10]. Studies have shown that patients who are taking non-steroidal anti-inflammatory drugs (NSAIDs) and/or corticosteroids routinely to address their chronic pain or illness also take PPIs to protect their stomachs [11]. Studies have also shown that in over 70% of cases, PPIs were frequently prescribed without a clear indication [9,12]. Without proper indication and guidance, the use of such medication may result in unwanted adverse side effects and could harm the patient.

Previous studies have shown that the persistent use of PPIs is associated with an increased risk of COVID-19 infection [13,14]. There is evidence that the regular use of PPIs could decrease the body’s natural gastric defense against ingested pathogens such as bacteria and viruses and alter the GI system’s microbial diversity [14,15]. There have been no data so far about the effect of acid suppression on SARS-CoV-2, although a previous study has shown that a pH level of 3, the normal pH of a healthy stomach, damages the infectivity of severe acute respiratory syndrome coronavirus 1 (SARS-CoV-1), a similar virus. However, it has been shown that the less acidic stomach pH attained through PPI therapy has failed to deactivate the virus [16]. Whether the use of PPIs increases a person’s susceptibility to SARS-CoV-2 infection or not remains unknown.

Previous studies have also shown varying results with regard to the effects that PPIs have on the severity and disease outcome of COVID-19. One study that focused on elderly people showed that PPIs had a protective effect when it comes to COVID-19 clinical manifestation [17]. This, however, was contradicted by two meta-analysis studies that linked PPI intake with poor COVID-19 outcomes, such as an increase in the mortality rate and severity of symptoms [15,18].

Various studies from a number of countries have confirmed that patients who were diagnosed with COVID-19 have manifested a variety of GI symptoms such as nausea, abdominal pain, and diarrhoea [19,20]. GI symptoms are of special significance among COVID-19 patients because, in contrast to other coronaviruses, they appear early and may worsen during the course of the disease, and in some cases may even appear as a solitary symptom [19]. There tends to be a delay in disease diagnosis among COVID-19 patients who present solely with GI symptoms, which renders these patients as potential sources of viral dissemination [19]. This fact alone highlights the importance of GI symptoms in COVID-19.

A review of previous studies has shown that these papers focused mainly on the effects that PPI consumption has on the severity and outcome of COVID-19 infection. The researchers could not find any literature on the effect of PPI use on the clinical presentation of patients with COVID-19, particularly on the GI manifestations of patients who were undergoing PPI therapy. The researchers sought to bridge this gap in the available literature by conducting this study, which aims to establish a possible relation between the regular use of PPIs and the disease presentation of COVID-19, particularly its GI manifestation.

## 2. Methods

### 2.1. Study design and data sources

This study was a clinical observational and cross-sectional cohort study conducted in a single tertiary care hospital in Amman, Jordan, after receiving approval from the Institutional Review Board. The study involved adult patients aged 18 years and above (age ≥18 years) who were admitted to the Prince Hamza Hospital for isolation and treatment of COVID-19. The study was conducted over a three-month period from May 6 to August 6, 2022. The study participants comprised patients who were laboratory-confirmed positive for SARS-CoV-2 through polymerase chain reaction (PCR) nasopharyngeal swab tests. All of the study subjects were asked to sign a consent form indicating that they had agreed to participate in the study. The study excluded patients who were uncooperative, those who were under the age of 18 years when they had their first symptom of COVID-19, patients who used PPIs only occasionally, and those who used acid suppression medications other than PPIs such as H2 blockers, and patients with previous upper-GI surgery. In addition, any patients with previous abdominal pain or diarrhea were also excluded.

Upon hospital admission, patients were interviewed in person with the help of a trained nurse who used a three-part predesigned questionnaire. The first part of the questionnaire covered demographic information such as sex, age, height, weight, history of comorbidities, alcohol consumption as well as tobacco use. Comorbidities included in the questionnaire were heart disease, lung disease, diabetes mellitus, renal disease, hypertension, hyperlipidemia, inflammatory bowel disease, thyroid disease, and GI diseases. Each patient’s body mass index was also calculated using self-reported height and weight information.

The second part of the questionnaire focused on the clinical presentations of COVID-19. Patients were asked if they experienced any of the following symptoms listed in the questionnaire during the course of their illness. The listed symptoms were divided into four parts as follows: GI symptoms (nausea, vomiting, diarrhoea, abdominal pain or discomfort, heartburn, jaundice, constipation), gustatory or olfactory symptoms (loss or change in smell, loss or change in taste), respiratory symptoms (cough, dyspnoea, sore throat, sputum production, rhinorrhea, insomnia, haemoptysis) and flu-like symptoms (fever, fatigue, myalgia, chills, arthralgia, headache, loss of appetite).

The third part of the questionnaire pertained to the patient’s history of PPI consumption, whether they took PPI regularly or not, and for how long. Patients were categorized into two groups based on their consumption of PPI drugs such as Pantoprazole, Omeprazole, Rabeprazole, Esomeprazole, and Lansoprazole. These were 1) PPI non-users: patients with COVID-19 infection who did not take any type of PPI before their infection; and 2) PPI users: patients with COVID-19 infection who had been taking any type of PPI for at least four weeks before their first COVID-19 presentation.

All of the research data were gathered with the patient’s consent and were anonymized to guarantee patient confidentiality. The patient clinical outcomes as well as confirmation of their PPI usage were obtained from the electronic patients’ medical records. Clinical outcomes included hospitalization in a ward or intensive care unit (ICU), the need for mechanical ventilation, and mortality.

### 2.2. Ethical approval

This study was granted ethical approval by the Hashemite University and the Prince Hamza Hospital’s Ethics Service Committee with reference number 5/3/2020/2021. All experimental protocols were approved by Hashemite University and the Prince Hamza Hospital’s Ethics Service Committee. All of the study participants provided written informed consent prior to their induction into the study. All the methods were carried out in accordance with the relevant guidelines and regulations.

### 2.3. Outcome measure

The primary outcome of the study was the assessment of any established association between the patients’ PPI consumption and their clinical presentation of COVID-19, particularly their GI manifestations.

### 2.4. Statistical analysis

The researchers used a Graph prism when performing the statistical analysis. Qualitative variables were described using absolute and relative frequencies, while quantitative variables were described using mean and confidence intervals. The Kolmogorov–Smirnov test was employed to assess the normality of data. The comparison of parametric quantitative normalized variables between two groups was performed using the independent sample *t*-test. Quantitative non-parametric variables were compared between the two groups by the Mann–Whitney U-test and between groups of more than two using the Kruskal–Wallis test. The Chi-square test was used to compare the qualitative variables. The researchers performed a multivariable logistic regression on reporting a positive COVID-19 test to adjust for a wide range of potentially confounding factors and to calculate adjusted odds ratios (ORs) and 95% confidence intervals (CIs). A 2-tailed *P* value < 0.01 was considered statistically significant.

## 3. Results

### 3.1. Patient characteristics

A total of 254 patients who were diagnosed with COVID-19 were enrolled in the study. Table 1 shows the detailed clinical characteristics of the subjects. The mean age of the patients was 59.7 ± 16.5 years, and 52.7% (*n* = 134) of the participants were males with ages ranging from 25 to 91 years while 47.2% (*n* = 120) were females. The majority of participants were overweight with a BMI mean of 27.9 ± 4.6. More than half or 68.1% (*n* = 143) of the study subjects were non-smokers, and 58% (*n* = 122) of the patients had at least one comorbid condition. The most common comorbid condition was cardiovascular disease.

**Table 1.** Baseline demographic, social and clinical characteristics of COVID-19 patient cohort.

Patient Characteristics	All patients with	Non-PPI user	PPI user	*P* value
	COVID-19 (*N* = 254)	(*n* = 185, 72.8%)	(*n* = 69, 27.2%)	
Age, y, mean ± SD	59.7 ± 16.5	59.2 ± 17.0	60.9 ± 16.4	0.16
Female, n (%)	120 (47.2%)	89 (48.1%)	31(44.9%)	0.67
BMI, kg/m^2^, mean ± SD	27.9 ± 4.6	27.5 ± 4.9	29.1 ± 3.3	<0.0001
**Past medical history, *n* (%)**				
Coronary artery disease	45 (17.7%)	33 (17.8%)	12 (17.3%)	0.36
Congestive heart failure	31 (12.2%)	24 (12.9%)	7 (10.0%)	0.40
Cardiac arrhythmia	25 (9.8%)	18 (9.7%)	7 (10.0%)	0.43
Hypertension	53 (20.8%)	39 (21.8%)	14 (20.2%)	0.33
Hyperlipidaemia	49 (19.3%)	34 (18.3%)	15 (21.7%)	0.59
Diabetes	50 (19.6%)	37 (20.0%)	13 (18.8%)	0.60
Cerebrovascular accident	20 (7.8%)	14(7.5%)	6 (8.6%)	0.79
Pulmonary disorders	44 (17.3%)	31 (16.7%)	13 (18.8)	0.71
renal disease	31 (12.2%)	23 (12.4%)	8 (11.5%)	0.41
Thyroid disorders	21 (8.2%)	14 (7.5%)	7 (10.0%)	0.60
Gastroesophageal reflux disease	57 (22.4%)	18 (9.7%)	39 (56.5%)	<0.0001
Irritable bowel syndrome	21 (8.1%)	16 (8.6%)	5 (7.2%)	0.80
Inflammatory bowel disease	8 (3.1%)	6 (3.2%)	2 (2.9%)	0.47
Peptic ulcer disease	38 (14.9%)	9 (4.8%)	29 (42.0)	<0.0001
**Social history, *n* (%)**				
Alcohol use	17 (6.7%)	12 (6.4%)	5 (7.2%)	0.78
Tobacco use	56 (22.0%)	39 (21.0%)	17 (24.6%)	0.61

The study participants were divided into two groups based on their PPI usage: users and non-users. Of the 254 participants, 69 had a history of PPI consumption and 185 (72.8%) were considered non-users. Some, meaning 8 of the 69 patients (11.5%), had a double-dose PPI consumption while the rest only had one dose in the early mornings.

The most frequently prescribed PPI was Esomeprazole, which was taken by 27.5% (*n* = 19) of patients, followed by Lansoprazole taken by 24.6% (*n* = 17), Pantoprazole taken by 21.7% (*n* = 15), Rabeprazole taken by 15.9% (*n* = 11), and Omeprazole taken by 10.0% (*n* = 7) of patients.

The study found no statistically significant difference between the two groups when it came to their age, gender, and social history which pertained to their tobacco and alcohol consumption. There was, however, a statistically significant difference between the two groups based on their BMI values, with the PPI users having a higher BMI mean value (*t* = 72.78, df = 68, *p* < 0.0001). With regards to comorbid conditions, the study found no statistically significant difference between the two groups except in cases of GERD and peptic ulcer disease, which were significantly higher among PPI users.

### 3.2. Clinical presentation of the participants

The most commonly reported symptoms among the study participants were fever (95.2%), followed by dyspnoea (96.4%), cough (91.7%), and fatigue (90.5%). Other reported symptoms included loss of appetite, which was experienced by 74.8% of participants, loss of smell (70.4%), and loss of taste (35.0%).

The study found that about a third or 33.5% of the study participants reported at least one GI symptom on presentation. The most commonly reported GI symptoms were diarrhoea (18.1%), followed by vomiting (17.3%), abdominal pain (17.3%) and constipation (10.0%), (Table 2). The study found that GI symptoms were not a predominant presenting complaint, nor the initial presenting symptoms of COVID-19 among all participants.

**Table 2.** Signs and symptoms reported by the participants.

Symptoms	All patients with	Non-PPI user	PPI user	*P* value
	COVID-19 (*N* = 254)	(*n* = 185, 72.8%)	(*n* = 69, 27.2%)	
**General symptoms**				
Fever	242 (95.2%)	179 (96.7)	63 (91.3)	0.25
Fatigue	230 (90.5)	171 (92.4)	59 (85.5)	**0.14**
Myalgia	115 (45.3)	96 (51.9)	19 (27.5)	**0.0006**
Chills	130 (51.1)	89 (48.1)	41 (59.4)	0.12
Arthralgia	159 (62.5)	120 (64.8)	39 (56.2)	0.24
Headache	77 (30.3)	55 (29.7)	22 (31.8)	
Loss of appetite	190 (74.8)	133 (70.2)	57 (82.6)	**0.10**
**Respiratory symptoms**				
Cough	233 (91.7)	169 (91.3)	64 (92.7)	0.59
Dyspnoea	245 (96.4)	179 (96.7)	66 (95.6)	**0.09**
Sore throat	126 (49.6)	89 (48.1)	37 (53.6)	**0.48**
Sputum production	74 (29.1)	52 (28.1)	22 (31.9)	0.64
Rhinorrhea	40 (15.7)	30 (16.2)	10 (14.5)	0.84
Loss of smell	179 (70.4)	128 (69.1)	51 (73.9)	**0.53**
Loss of taste	89 (35.0)	64 (34.5)	25 (36.2)	**0.26**
Anosmia	85 (33.5)	60 (32.4)	25 (44.9)	**0.65**
Haemoptysis	15 (5.9)	12 (6.4)	3 (4.3)	**0.43**
**Gastrointestinal symptoms**				
Any GI symptoms	85 (33.5%)	61 (33.0)	24 (34.8)	**0.88**
Diarrhoea	46 (18.1)	26 (14.1)	20 (28.9)	**0.02**
Nausea	43 (16.9)	30 (16.8)	13 (18.8)	**0.36**
Vomiting	44 (17.3)	32 (17.2)	12 (17.4)	**0.35**
Abdominal pain	44 (17.3)	25 (13.5)	19 (27.5)	**0.01**
Heartburn	28 (11.0)	24 (12.9)	4 (5.7)	**0.03**
Constipation	26 (10.2)	18 (9.7)	8 (11.6)	**0.64**
Jaundice	0	0	0	**0**

Compared to non-PPI users, patients who were on PPI treatment reported significantly lower rates of myalgia (27.5% vs 51.9%; *p* = 0.0006) and heartburn (5.7% vs 15.6%; *p* = 0.03) but significantly higher rates of abdominal pain (27.5% vs 13.5%; *p* = 0.001) and diarrhea (28.9% vs 14.5%, *p* = 0.02).

The most commonly reported GI symptoms among non-PPI users were nausea, vomiting, and diarrhoea, whereas the most commonly reported GI symptoms among those who were on PPI treatment were abdominal pain, diarrhoea, and nausea.

The study also found that flu-like symptoms were less common among PPI users, except for chills which were reported more among this group. However, the difference was not statistically significant between the two.

With regard to respiratory symptoms, the study found no significant difference between the PPI user and non-PPI user groups. While there was a higher reported incidence of symptoms such as cough, sore throat, sputum production, loss of smell, and loss of taste among the PPI user group, the difference was not statistically significant.

### 3.3. PPI use and patient clinical outcomes

Among the cohort of patients, 25 (9.6%) individuals needed admission to the ICU while 14 (5.3%) people required mechanical ventilation (Table 3). Altogether, a total of seven (2.6%) in-hospital deaths were recorded. The study found no statistically significant differences in the rates of clinical deterioration between patients who were with and without PPI treatment in terms of ICU admission, the need for mechanical ventilation, or overall mortality, although these were higher among the PPI user group.

**Table 3.** Clinical outcomes of the participants in this study related to ICU admission, the need for mechanical ventilation, and mortality.

	All patients with	Non-PPI user	PPI user	*P* value
	COVID-19 (*n* = 254)	(*n* = 185, 2.8%)	(*n* = 69, 27.2%)	
ICU stay	25 (9.6%)	17 (9.1%)	8 (11.6%)	0.63
Mechanical ventilation	14 (5.3%)	9 (4.8%)	5 (7.2%)	0.53
Death	7 (2.6%)	4 (2.1%)	3 (4.3%)	0.39

### 3.4. Association between PPI and clinical presentation

**Figure 1.**
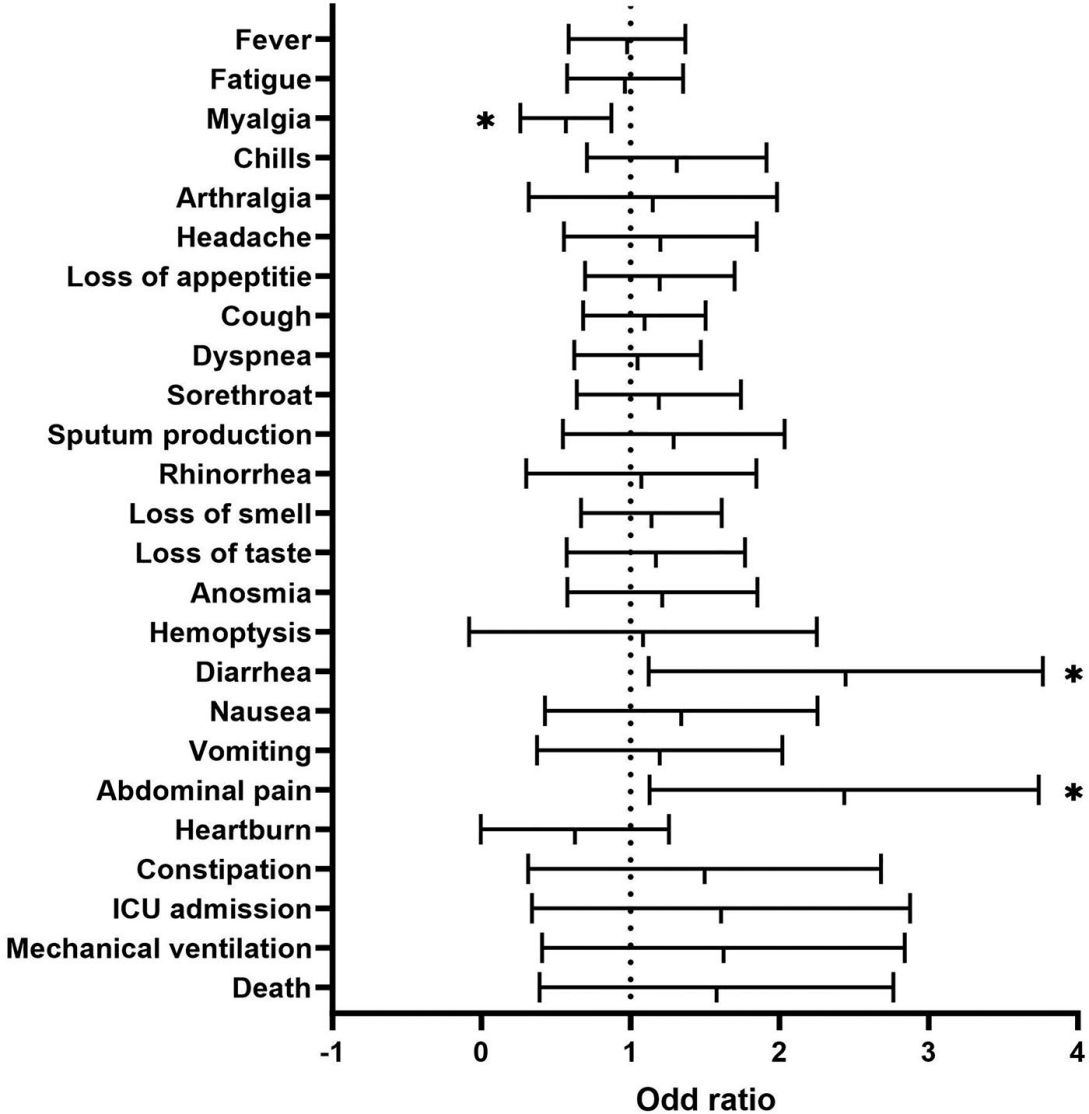
Forest plots showing Adjusted odds ratio (OR) and 95% confidence interval (CI)(X-axis) of developing different signs and symptoms (Y-axis) associated with the use of PPI among COVID-19 disease. * statistically significant.

A multivariable regression analysis was conducted across the full sample with controls set for age, gender, BMI, comorbidity, and history of smoking. When compared to individuals who were not on PPI therapy, those who were taking PPIs showed significantly increased odds of developing diarrhoea (OR 2.0, 95% CI: 1.08 to 3.93, *p* = 0.02) and experiencing abdominal pain (OR 2.0, 95% CI: 1.22 to 3.93, *p* = 0.03) but had a lower risk of developing myalgia (OR 0.5, 95% CI: 0.3 to 0.9, *p* = 0.02) (Figure 1).

## 4. Discussion

PPIs are one of the most commonly prescribed medications for the treatment and prevention of acid-related GI diseases in the world today [21]. The long-term consequences of PPIs have come under scrutiny due to their increased use over the past 20 years [22]. Numerous research has examined the relationship between the use of PPIs and several types of infection, namely *Clostridium difficile* and pneumonia [23]. It is hypothesized that PPI-induced changes to the gut microbiota foster the growth of certain kinds of infections [24].

PPI’s potential to contribute to the onset or advancement of chronic renal disease is one of the more recent issues surrounding the drug [25]. Numerous long-term adverse effects have also been studied, including drug interactions, liver and cardiovascular disease, enteroendocrine tumors of the gastrointestinal tract, decreased intestinal absorption of vitamins and minerals, and, more recently, dementia and kidney damage [21,22,26].

A number of studies have been conducted on the impact of PPIs on the severity and outcome of COVID-19 infection, but none has drawn attention to the effects of PPIs on the clinical presentation of COVID-19 infection. This present study was undertaken to assess and determine a potential link between PPI use and the development of GI and respiratory symptoms in patients with COVID-19 infection.

In this present observational cross-sectional study, PPI consumption was found to be higher in men and older patients, and the most frequently prescribed PPI was Esomeprazole at 27.5%. The study also showed that some COVID-19 symptoms particularly some GI manifestations occurred in higher proportions among PPI users than in non-PPI users. This could be due to a number of reasons, such as a more severe baseline GI disease for which the PPIs were prescribed, the prolonged use of PPI leading to side effects, or the survival ability of the SARS-CoV-2 virus and its impact on the GI tract.

PPIs work by suppressing the output of gastric acid through the irreversible mechanism of proton pump inhibition [27]. This results in profound hypochlorhydria or low stomach acid which weakens the protective effect of gastric acid. This in turn increases the chance of survival of the SARS-CoV-2 virus in the stomach and facilitates its invasion of enterocytes [28,29]. In addition, the reduction in stomach acid production due to PPI consumption can alter the makeup of the gut’s microbiome and could lead to more prevalent enteric infections. This goes to show that PPI use can leave the gut more susceptible to coronavirus infection.

According to a study by Xiao, Chakraborti [30], the S protein of SARS-CoV facilitated the fusion of the virus with host cells under neutral pH conditions. Darnell, Subbarao [16] confirmed in their study that extremely alkaline (pH 12 and 14) and highly acidic (pH 1 and 3) conditions can lead to the deactivation of the SARS-CoV virus, although the virus can remain stable within a range of neutral pH levels. A similar study showed SARS-CoV-2 remaining alive on the sixth day but losing between 2.9 and 5.33 logs of infectivity at pH levels of 5–9. At extreme pH levels of pH 2–3 and pH 11–12, SARS-CoV-2 lost infectivity within a day [31]. Another study by Zhou, Niu [32] showed that the SARS-CoV-2 virus was completely inactivated and incapable of infecting cells under highly acidic conditions of pH 1.0 and 2.0, similar to the normal acidity of an empty stomach, by making viruses pseudotyped with SARS-CoV-2 S protein.

Stomach secretions have a pH level between 1.0 and 3.5 while the small and large intestines have a pH level between 7.5 and 8.0. Normally, gastric acid has the ability to neutralize most virus particles such as those of the SARS-CoV-2 virus. However, if a person takes an acid suppressant such as a PPI medication for a long period of time, their gastric condition becomes less acidic. In this case, the SARS-CoV-2 virus attains a greater chance of entering the gut from the stomach, leading to a viral infection and a higher risk of developing a GI manifestation. This study showed that patients who used PPIs exhibited significantly higher incidences of abdominal pain and diarrhoea.

In the presence of COVID-19 infection, PPI administration may result in diarrhoea and abdominal pain through a number of different mechanisms, such as facilitated enteropathogenic bacterial colonization, insufficient stomach protein digestion, and potential effects on H, K-ATPase in the colonic mucosa [33,34]. Certain types of bacteria, such as the *Clostridium difficile*, *Salmonella*, and *Campylobacter* species, have been shown to readily grow in the gut in the absence of stomach acid, leading to enterocolitis that is accompanied by diarrhoea or loose stools [35,36]. Moreover, PPI-induced disruption of the stomach acid barrier results in small intestine bacterial overgrowth (SIBO). SIBO symptoms are non-specific and include diarrhoea and soreness or discomfort in the abdomen.

Digestive symptoms such as an altered sense of smell and taste, nausea, vomiting, loss of appetite, and diarrhea are quite common among patients with COVID-19, and we found that these symptoms were more common among PPI users. Several studies have described worse clinical outcomes among COVID-19 patients who were also manifesting GI symptoms [37,38].

As the COVID-19 pandemic happened, a number of studies were carried out to find a potential causative, susceptibility, prognostic, or preventive effect that PPI usage may have on COVID-19. One study showed a direct correlation between PPI consumption and a more severe COVID-19 infection with worse clinical outcomes [15]. Other meta-analyses demonstrated that PPI use was not associated with a higher risk of infection and mortality among COVID-19 patients. However, an association between PPI usage and a higher risk of developing a more severe presentation of the disease and a higher risk of developing a secondary infection was found [18]. This was corroborated by this present study, which showed the same results wherein a higher percentage of patients who took PPIs were admitted to an ICU (*p* = 0.6) and required mechanical ventilation (*p* = 0.5). Our study found a mortality rate of 4.3% among PPI users and 2.1% among non-PPI users (P-value = 0.39). The researchers hypothesized that this could be related to the development of a secondary infection that was triggered by PPI use [39]. Furthermore, the authors recommended that further studies are needed to clarify the association between PPI consumption and COVID-19 infection and its consequences.

It is a well-known fact that COVID-19 affects the elderly more than any other population group. It has been shown that the gastric pH level in many older adults is considerably higher than normal, which scientists think could be due to atrophic gastritis or the use of acid-reducing medications [40]. Children, on the other hand, have largely normal gastric pH levels which offer them some kind of protection from a severe infection of COVID-19 [41,42]. The researchers believe these factors could play a role in part in evaluating people’s susceptibility to COVID-19 infection. The link between a higher gastric pH level and greater colonization of gastric microbiomes has been proven in many studies [43]. Several studies have suggested that a higher viral load can lead to a more symptomatic and severe COVID-19 infection [44,45]. It appears that a higher load of COVID-19 virus can induce a stronger cytokine storm and more colonization by the virus, resulting in a more severe presentation of COVID-19 [44,45].

The authors of this study believe this to be the first study to examine the relationship between PPIs and GI manifestation among COVID-19 patients. None of the previous studies have assessed the impact of PPI use on the clinical presentation of COVID-19. Unlike many studies to examine the potential impact that PPIs have on the severity of COVID-19 patients, the researchers of this current study constructed this study using a predesigned questionnaire and followed up with the patients during the whole duration of their stay in the hospital. However, this study has some limitations. For example, patients may have used over-the-counter antacids and other acid-reducing drugs which were not reported in this study, resulting in reporting bias. The results of single-site studies like this one, which has a limited sample size of patients presenting with COVID-19, may be less generalizable or widely applicable to other settings and populations. Despite these limitations, the findings of this study highlighted that patients’ use of some medication, including PPIs prior to hospitalization, is important and could impact the outcome and clinical presentation of COVID-19. Therefore, the findings of this study could provide a vital roadmap for future studies to evaluate the effects of other acid suppression medications, as well as other medications for gastrointestinal disorders on the clinical presentation of COVID-19. In addition, many intubated patients were given acid-reducing drugs. Such practices could result in a gastric pH level of around 4.0 or 5.0. This would not incapacitate viruses like the SARS-CoV-2 virus, which could then pass into the small intestines where the ACE2 receptors are abundant. It is therefore recommended that if there is evidence that gastric acidity offers some protection, discontinuing the use of antacids and acid-reducing medications should be considered, especially at times when patients are at their most vulnerable.

The authors of this study have reached the conclusion that the effects of PPI use in relation to the clinical presentation of COVID-19 merit further investigation in different populations and settings. The findings of this study show that PPIs could impact the gastrointestinal systems of patients with COVID-19, which could lead to different clinical presentations and outcomes. Further studies are needed to evaluate whether there is a correlation between PPI use and indicators of COVID-19 severity such as hospitalization, the need for intubation, and mortality. Widespread strategies should also be considered to promote rational prescription of PPIs and prevent the misuse of PPIs, especially among high-risk groups of the population.

## Data Availability

The datasets used and/or analysed during the current study are available from the corresponding author on reasonable request.
